# Tumor suppression by p53 without apoptosis and senescence: conundrum or rapalog-like gerosuppression?

**DOI:** 10.18632/aging.100475

**Published:** 2012-07-31

**Authors:** Mikhail V. Blagosklonny

**Affiliations:** Department of Cell Stress Biology, Roswell Park Cancer Institute, Buffalo, NY 14263, USA

**Keywords:** tumor suppressors, aging, apoptosis, geroconversion

## Abstract

I discuss a very obscure activity of p53, namely suppression of senescence (gerosuppression), which is also manifested as anti-hypertrophic, anti-hypermetabolic, anti-inflammatory and anti-secretory effects of p53. But can gerossuppression suppress tumors?

## INTRODUCTION

Wt p53 can induce apoptosis, cell cycle arrest and senescence, which are sufficient to explain tumor suppression by p53 [1]. A recent paper in *Cell* described that these activities are dispensable for tumor suppression [2]. Mutant p53 (p53^3KR^) that cannot cause arrest, senescence and apoptosis still suppressed tumors in mice [2, 3]. Why do then wt p53 induce apoptosis, cell cycle arrest and senescence? Before entertaining this intriguing question, I will focus on suppression of senescence (gerosuppression) by p53, overlapping with its anti-hypertrophic, anti-hypermetabolic, anti-inflammatory and anti-secretory effects.

### P53 suppresses the conversion from arrest to senescence (geroconversion)

How can p53 suppress senescence, if it also can cause senescence? As recently suggested, induction of senescence is not an independent activity of p53 but a consequence of cell-cycle arrest [4-8]. This predicts that any mutant p53 that cannot cause arrest will not cause senescence too. In agreement, p53^3KR^ did not cause senescence [2]. This is not trivial. To create p53^3KR^, wt p53 was altered to abolish apoptosis and cell-cycle arrest only [2]. Li et al did not modify p53 to abolish senescence as an independent activity. It was not needed, simply because p53 does not induce senescence as an independent effect. (Note: Seemingly in contrast, it was reported that mutant p53, which cannot induce arrest in response to DNA damage, can cause senescence [9]. Although this mutant p53 did not cause instant arrest, it still arrested proliferation later and then senescence developed [9]. So there is no exception). p53 cannot induce senescence without inducing arrest. But p53 can induce quiescence, a reversible condition characterized by low protein synthesis and metabolism (see detailed definitions in ref. [7, 8]). It was assumed that when p53 causes quiescence, it simply fails to induce senescence. But another possibility is that in such cases p53 suppresses the conversion from cell-cycle arrest to senescence (geroconversion). How can that be tested? In some cell lines, induction of ectopic p21 causes irreversible senescence, whereas induction of p53 causes quiescence [4]. Does p53 suppresses a senescent program? This question can be answered by simultaneously inducing both p53 and ectopic p21. When both p21 and p53 were induced, then cells become quiescent not senescent [4]. p53 was dominant, actively suppressing senescence caused by p21… or by something else? In fact, p21 merely causes cell cycle arrest and does not inhibit mitogen-activated, nutrient-sensing and growth-promoting pathways such as Target of Rapamycin (mTOR) [4]. During several days, these pathways (gerogenic pathways, for brevity) convert p21-induced arrest into senescence. Rapamycin can decelerate geroconversion [10-13]. Also, p53 can inhibit the mTOR pathway [4-6, 14-17]. In some conditions, p53 can suppress senescence during arrest [4-6]. Wt p53 induces arrest and then if it fails to suppress senescence, then senescence prevails. Rather than p53, gerogenic pathways drive senescence during cell-cycle arrest [18].

In summary, wt p53 seems to have three independent effects: apoptosis, cell-cycle arrest and gerosuppression. By inducing arrest, wt p53 primes cells for senescence, unless p53 is able or “willing” to suppress geroconversion. At high levels, gerosuppression by p53 is limited by apoptosis [6]. This predicts that p53^3KR^ would potently suppress senescence because gero-suppression by p53^3KR^ will not be limited by apoptosis.

### Hyper-metabolic senescent phenotype

**Figure 1:**
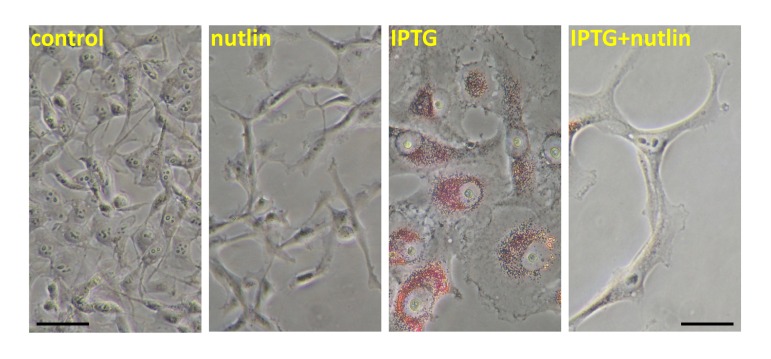
Nutlin-3a decreased lipid accumulation during IPTG-induced senescence HT-p21 cells were treated with IPTG, nutlin-3a and IPTG+nutlin-3a (as indicated) for 3 days as described previously [4-6] and cells were stained with “oil red O” for lipids. In HT-p21 cells, IPTG induces ectopic p21 and senescence. As described previously, nutlin-3a induces endogenous p53 and suppresses IPTG-induced senescence [4-6].

Senescent cells are hyper-functional: hypertrophic, hypermetabolic, hyper-secretory and hyper-inflammatory [8]. Also, senescent cells may accumulate lipids, becoming not only large but also “fat” (Figure1). Induction of p53 decreased both cellular hypertrophy and fat accumulation (Figure 1). This is in line with numerous metabolic effects of p53 including inhibition of glycolysis and stimulation of fatty acids oxidation [19-32]. Importantly, p53^3KR^ retained the ability to inhibit glycolysis and reactive oxygen species (ROS) [2]. (Noteworthy, ROS and mTOR co-activate each other [33] and N-Acetyl Cysteine (NAC), which decreases ROS, also inhibits mTOR [34]). Also, p53 decreases hyper-secretory phenotype also known as SASP [35] and suppresses a pro-inflammatory phenotype [36, 37]. How might gerosuppression contribute to tumor suppression? There are several overlapping explanations, from different points of view of the same process.

### Gerogenic conversion and oncogenic transformation

In proliferating epithelial cells, pro-gerogenic conversion may contribute to carcinogenesis directly. The PI3K/mTOR pathway is universally activated in cancer [38-49]. p53 can inhibit the PI3K/mTOR pathway [4-6, 14-17, 50]. Like p53, many other tumor suppressors such as PTEN, AMPK, TSC2, LKB1, NF1 inhibit the PI3K/mTOR pathway [51].

### Geroconversion of stromal cells creates carcinogenic microenvironment

First, senescence creates a selective disadvantage for normal cells, thus selecting for cancer [52-54]. Also, senescent stromal cells secrete factors that favors pre-cancer and cancer growth [37, 54-62]. Third, the senescent stroma is hyper-metabolic and thus promotes cancer by fueling cancer growth [59, 60, 63-71]. In a model of accelerated host aging, mTOR activity was increased in normal tissues [72]. This pro-senescent microenvironment accelerated growth of implanted tumors. The tumor-promoting effects of pro-senescent microenvironment were abrogated by rapamycin [72].

### Cancer is an age-related disease

The incidence of cancer is increased exponentially in aging mammals. Manipulations that slow down aging delay cancer [73]. For example, calorie restriction delays cancer [74-76] including cancer in p53-deficient mice [77, 78]. Rapamycin, which decelerates aging, also postpones cancer in animals [73, 79-81] and in patients after renal transplantation [82-86].

### Is aging accelerated in p53-deficient mice?

Inactivation of tumor suppressors accelerates both aging and cancer [87]. It was thought that p53 is an exception. Yet, given that p53 can suppress geroconversion, it may not be the exception after all. A complex role of p53 in cellular senescence and organismal aging was discussed [88-91]. Mice with increased, but normally regulated, p53 lives longer [92]. p53 knockout mice have both accelerated carcinogenesis and decreased longevity [93-98]. p53−/− mice have a pro-inflammatory phenotype characteristic of accelerated aging [36, 37]. Also, atherosclerosis is accelerated in p53−/− animals [99-102]. While loss of p53 by itself makes cells prone to become tumorigenic, an increased rate of organismal aging in the absence of p53 may further accelerate carcinogenesis.

### Rapalogs and p53

Rapamycin (sirolimus) and other rapalogs (everolimus and temsirolimus) are pharmacological tumor suppressors. Noteworthy, like p53, rapamycin decreases glycolysis [103] and lactate production [34] and stimulates oxidation of fatty acids [104, 105]. Furthermore, rapamycin slows cellular proliferation, and so, not surprisingly, p53^3KR^ inhibits clonogenicity too [2]. Yet, p53 affects metabolism and aging not only via mTOR but also via direct transactivation of metabolic enzymes, rendering it a more potent tumor suppressor.

### Puzzles remain

Still, even if gerosuppression and anti-hypermetabolic effects can in part explain tumor suppression, puzzles remain. Why does wt p53 cause “unneeded” apoptosis and “instant” (p21-dependent) arrest? Why is p53 needed at all? In the wild, most mice die from external/accidental causes and only a few would live long enough to die from cancer, regardless of p53 status. In the wild, starvation (natural calorie restriction) would delay cancer further. Yet, p53 is also needed very early in life, or technically speaking, even before life has begun, because p53 plays role in fertility and reproduction [106-113]. And is tumor suppression a late life function?

Alternatively, tumor suppression is a primary function of p53. And each of the three activities (apoptosis, arrest, gerosuppression) is partially sufficient for cancer prevention. In their combination, these activities are the most effective tumor suppressor. And each activity may be partially dispensable in some mice strains and in some conditions. For example, the gerosuppressive activity of p53 may be preferentially important in peculiar strains of laboratory mice, or mice fed *ad libitum*, which constantly activates mTOR and accelerates aging. In fact, calorie restriction, which deactivates mTOR and decelerates aging, partially substitutes for the loss of p53 in mice.
